# Notch1 signaling regulates the epithelial–mesenchymal transition and invasion of breast cancer in a Slug-dependent manner

**DOI:** 10.1186/s12943-015-0295-3

**Published:** 2015-02-03

**Authors:** Shan Shao, Xiaoai Zhao, Xiaojin Zhang, Minna Luo, Xiaoxiao Zuo, Shangke Huang, Ying Wang, Shanzhi Gu, Xinhan Zhao

**Affiliations:** The Department of Oncology, the First Hospital Affiliated to the School of Medicine, Xi’an Jiaotong University, 277 West Yanta Road, Xi’an, 710061 Shaanxi Province China; The Department of Forensic Medicine, Medical School, Xi’an Jiaotong University, 76 West Yanta Road, Xi’an, 710061 Shaanxi Province China

**Keywords:** Breast cancer, Notch1 signaling, Invasion, Epithelial-mesenchymal transition, Slug

## Abstract

**Background:**

The epithelial–mesenchymal transition (EMT) is crucial for the invasion and metastasis of breast cancer. However, how Notch signaling regulates the EMT process and invasion in breast cancer remains largely unknown.

**Methods:**

The impact of Notch1 silencing by specific shRNAs on the EMT and invasion of human breast cancer MCF-7 and MDA-MB-231 cells as well as xenografts was tested by western blot, real-time polymerase chain reaction (RT-PCR), immunofluorescence, transwell, and immunohistochemistry assays. The effect of Slug silencing or upregulation on the EMT and invasion of breast cancer cells was analyzed, and the effect of Notch1 signaling on Slug expression was determined by the luciferase reporter assay.

**Results:**

The Notch1 intracellular domain (N1ICD) and Jagged1 were expressed in breast cancer cells. Notch1 silencing reversed the spontaneous EMT process and inhibited the migration and invasion of breast cancer cells and the growth of xenograft breast cancers. The expression of N1ICD was upregulated significantly by Jagged1-mediated Notch signaling activation. Moreover, Jagged1-mediated Notch signaling promoted the EMT process, migration, and invasion of breast cancer cells, which were abrogated by Notch silencing. Furthermore, the N1ICD positively regulated the Slug expression by inducing Slug promoter activation. Importantly, the knockdown of Slug weakened the invasion ability of breast cancer cells and reversed the Jagged1-induced EMT process with significantly decreased expression of vimentin and increased expression of E-cadherin. In addition, Slug overexpression restored the Notch1 knockdown-suppressed EMT process.

**Conclusions:**

Our novel data indicate that Notch signaling positively regulates the EMT, invasion, and growth of breast cancer cells by inducing Slug expression. The Notch1–Slug signaling axis may represent a potential therapeutic target for breast cancer therapy.

**Electronic supplementary material:**

The online version of this article (doi:10.1186/s12943-015-0295-3) contains supplementary material, which is available to authorized users.

## Background

Breast cancer is one of the most frequently diagnosed cancers and the leading cause of cancer death in females worldwide. It accounts for 23% of total cancer cases and 14% of cancer deaths [1]. Although there have been substantial advances in the treatment of localized malignancies, metastatic breast cancer still lacks effective treatment and remains the primary cause of breast cancer mortality [2]. Mortality is almost invariably due to metastasis [2], which is a complex process involving a succession of cell biological events [3].

The process of epithelial–mesenchymal transition (EMT) plays a key role in tumor metastasis [4]. Many factors can stimulate the EMT process, during which epithelial cells lose their polarity and cell–cell contacts, acquire a migratory mesenchymal phenotype, and increase resistance to chemotherapy and radiotherapy [5,6]. The process of EMT coincides with the loss of epithelial markers, such as E-cadherin and occludin, and acquisition of mesenchymal markers, such as N-cadherin and vimentin [7]. E-cadherin is a repressor of cancer invasion and EMT induction [8,9]. A group of transcription factors has been demonstrated to be capable of orchestrating EMT in cancer progression. Snail, Slug, and ZEB2/SIP1 are direct transcriptional repressors of E-cadherin, whereas Twist and ZEB1 act indirectly on E-cadherin [10].

The zinc-finger transcription factors Slug (Snail2) and Snail (Snail1) belong to the Snail superfamily [11]. Slug and Snail are two known important transcription factors that initiate EMT through downregulation of E-cadherin expression in breast cancer, and their expression has been shown to be regulated by Notch signaling [12]. Interestingly, Slug expression presents a much stronger correlation with loss of E-cadherin in breast cancer cell lines than Snail expression, indicating that Slug is a likely *in vivo* repressor of E-cadherin expression in breast cancer [13].

Accumulating evidence demonstrates that Notch signaling regulates many physiological processes, including cell fate determination in the process of embryonic development, tissue maturity, tumor cell proliferation, cancer stem cell maintenance, EMT, and chemoresistance [14,15]. Notch receptors and ligands are single-pass transmembrane proteins that regulate cell fate via cell–cell contact [16,17]. Human Notch families have four receptors (Notch1–4) and five ligands (Delta-like-1, Delta-like-3, Delta-like-4, Jagged1, and Jagged2) [14]. Notch signaling is activated by ligand–receptor interactions between neighboring cells, promoting γ-secretase-dependent cleavage of the Notch receptor and releasing the Notch intracellular domain (NICD) into the nucleus, where the NICD binds to the transcription factor CSL, resulting in activation of the pathway [16,18]. The NICD/CSL complex causes the expression of target genes, such as those of the Hairy enhancer of spit (Hes) family.

The Notch signaling pathway is dysregulated in many human malignancies. Overexpression of Notch receptors and their ligands has been found in cervical, colon, head and neck, lung, and renal carcinoma; pancreatic and breast cancer; as well as acute myeloid, Hodgkin, and large-cell lymphomas [19-21]. The first evidence that Notch receptors are breast oncogenes was provided in mouse studies in which active forms of Notch1 or Notch4 formed spontaneous murine mammary tumors *in vivo* [22]. Moreover, overexpression of Notch1 and/or its ligand Jagged1 is related to the poorest overall patient survival in human breast cancer [23-25]. Accumulating evidence indicates that Notch1 cross-talk with other major cell growth and apoptotic regulatory pathways regulates the activity of transcription factors, such as nuclear factor kappa B (NF-κB) [26]. However, the role of Notch signaling in regulating EMT remains largely unknown.

In the current study, we found that Notch1 knockdown in breast cancer cells suppressed the EMT process, tumor growth, migration, and invasion using *in vitro* and *in vivo* models. Jagged1-mediated Notch signaling activation was able to activate the EMT process and increase migration and invasion in breast cancer mainly though upregulation of N1ICD, rather than Notch2 NICD (N2ICD), Notch3 NICD (N3ICD), or Notch4 NICD (N4ICD). Moreover, we revealed that Notch1 signaling played a vital role in regulating EMT mainly in a Slug-dependent manner. Our findings indicate that Notch1 signaling is a promising therapeutic target for preventing breast cancer progression.

## Results

### Notch1 and Jagged1 are expressed in human breast cancer cell lines

**Figure 1:**
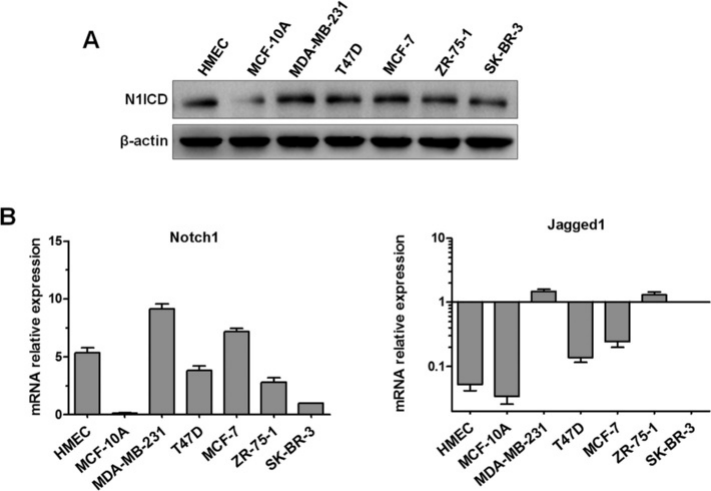
**The expression of Notch1 and Jagged1 in human breast cancer cell lines. (A)** The protein expression of N1ICD in a panel of breast cancer cells (MDA-MB-231, T47D, MCF-7, ZR-75-1, and SK-BR-3), HMECs, and MCF-10A cells was evaluated by western blot. Protein samples (150 μg) were separated by 10% SDS-PAGE. β-Actin was used as a loading control. **(B)** The mRNA expression of Notch1 and Jagged1 was estimated by real-time PCR. GAPDH was used as a normalization control for quantifying the expression of each target gene. Experiments were performed three times. Column: mean; bar: SD.

The NICD plays an important role in Notch signaling activation. To investigate the possible role of the Notch signaling pathway in initiation of the EMT process in breast cancer cells, we first explored the expression levels of the NICD of Notch1 (N1ICD) and its ligand Jagged1 in five human breast cancer cell lines, human mammary epithelial cells (HMECs), and non-tumorigenic MCF-10A cells. As shown in Figure 1A, the expression levels of the N1ICD protein were readily detectable in all seven cell lines by western blot. In addition, the Notch1 and Jagged1 mRNA levels were tested by real-time polymerase chain reaction (PCR) (Figure 1B). Based on the above results, MCF-7 and MDA-MB-231 cells were used to study the Notch1/Jagged1 signaling pathway.

### Stable Notch1 knockdown and Jagged1 were applied to inhibit or activate Notch signaling, respectively, in breast cancer cells

**Figure 2:**
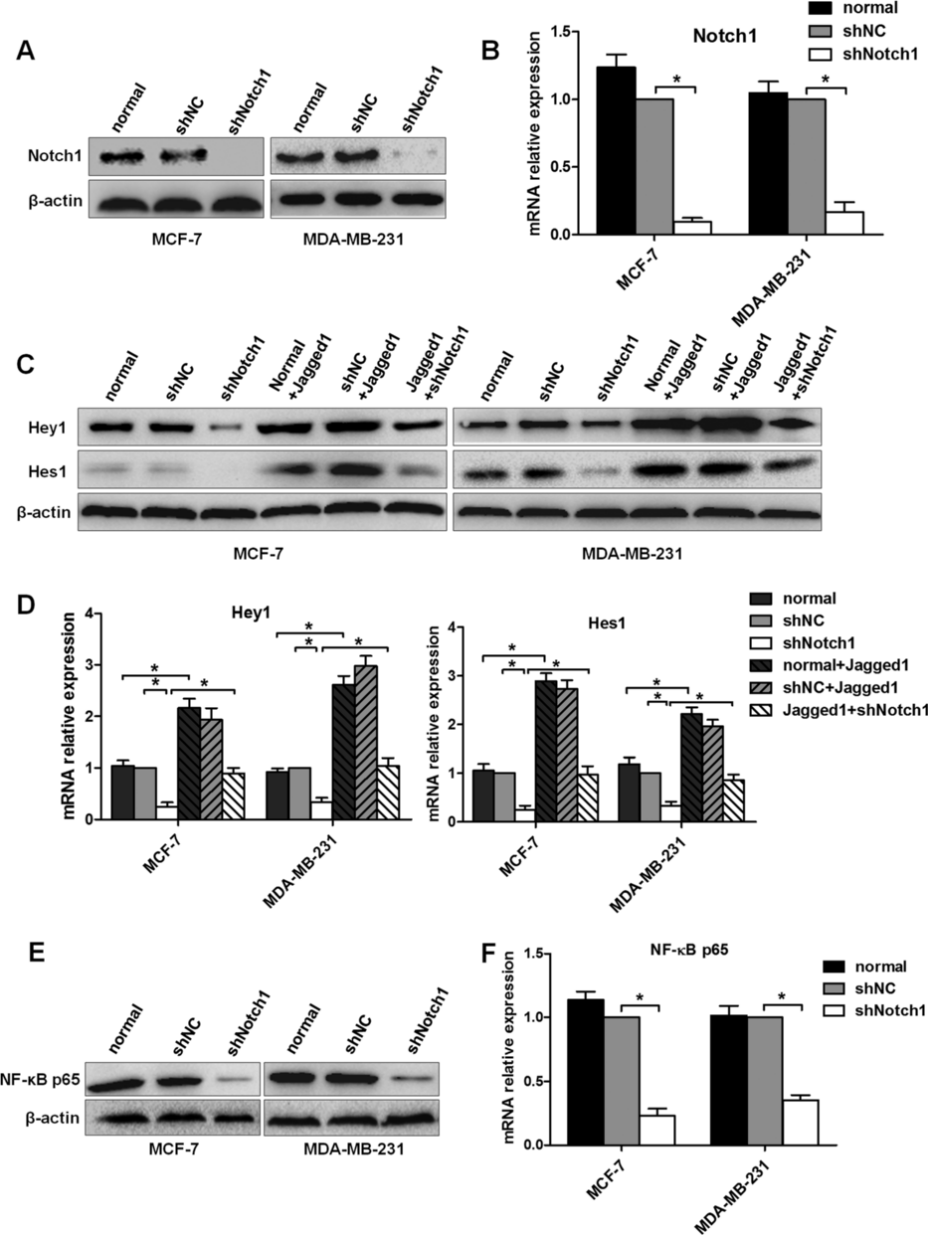
**Inhibition and activation of Notch signaling, respectively, in both MCF-7 and MDA-MB-231 cells.** Normal group: MCF-7 or MDA-MB-231 cells were incubated under normal conditions; shNC group: MCF-7 or MDA-MB-231 cells were stably transfected with NC shRNA; shNotch1 group: MCF-7 or MDA-MB-231 cells were stably transfected with Notch1 shRNA; normal + Jagged1: MCF-7 or MDA-MB-231 cells were treated with Jagged1 for 48 h; shNC + Jagged1 group: MCF-7 or MDA-MB-231 cells were treated with Jagged1 for 48 h after stable shNC transfection. Jagged1 + shNotch1 group: MCF-7 or MDA-MB-231 cells were treated with Jagged1 for 48 h and then Notch1 shRNA was transiently transfected for an additional 48 h. **(A)**, **(C)**, and **(E)** Total protein was isolated for western blot analysis using Notch1, Hey1, Hes1, and NF-κB65 antibodies. β-Actin was used as a loading control. **(B)**, **(D)**, and **(F)** Total RNA was extracted, and the expression levels of Notch1, Hey1, Hes1, and NF-κB65 were assayed by real-time PCR. The expression of every target gene was quantified using GAPDH as a normalization control. The data are from three independent experiments. Column: mean; bar: SD. The symbol * represents a significant difference (P < 0.05).

To investigate the role of Notch1 in Notch signaling, we established stable shRNA-transfected cells termed as MCF-7-shNotch1 and MDA-MB-231-shNotch1 (containing the Notch1 shRNA lentiviral vector), MCF-7-shNC, and MDA-MB-231-shNC (containing the negative control shRNA lentiviral vector). Western blot and real-time PCR analyses showed that, compared with shNC cells, the expression levels of Notch1 were inhibited up to 80–90% in both shNotch1 cell lines (P < 0.05, Figure 2A–B). Next, we examined the expression levels of Hey1 and Hes1, downstream genes of the Notch signaling pathway. Consistent with the reduction of Notch1, the expression levels of these genes were significantly decreased in both MCF-7 and MDA-MB-231 cells (Figure 2C–D). Previous studies have shown that Notch signaling strongly regulates the activity of its downstream gene NF-κB, while NF-κB has been identified to play an important role in the EMT process [26,27]. Therefore, we evaluated the expression of NF-κB p65, a NF-κB subunit. Our results showed that NF-κB p65 was downregulated by Notch1 knockdown in both MCF-7 and MDA-MB-231 cells (Figure 2E–F).

To explore the effect of Notch signaling, Notch signaling was activated by using an immobilized recombinant Jagged1-Fc chimera in MCF-7 and MDA-MB-231 cells. These two cell lines express multiple Notch receptors, thus providing cells with the capability to activate the Notch signaling pathway through ligand–receptor interactions. After treatment with Jagged1 for 48 h, we detected NICD expression of the four Notch receptors (Notch1, Notch2, Notch3, and Notch4). As shown in Additional file 1: Figure S1, both MCF-7 and MDA-MB-231 cells showed an increased expression of N1ICD and N4ICD. However, N2ICD and N3ICD were not significantly changed. Moreover, the expression of N1ICD was increased more than that of N4ICD, indicating that Notch1 may have a dominant role in Jagged1-mediated Notch signaling activation. Furthermore, the expression levels of Hey1 and Hes1 were significantly increased in both Jagged1-treated cell lines, compared with the normal group (Figure 2C–D). Interestingly, the downregulation of Notch1 could only partially abolish the effect of Jagged1-induced activation of Notch signaling on Hey1 and Hes1 (Figure 2C–D). These results indicate that Notch4 may be an alternate pathway to regulate Jagged1-induced Hey1 and Hes1 expression, but this effect may be much weaker than Notch1 as Notch1 knockdown suppressed most of the Hey1 and Hes1 expression induced by Jagged1. Thus, our data indicate that Hey1, Hes1, and NF-κB p65 are mainly regulated by Notch1 signaling.

### Notch1 knockdown reverses the Jagged1-induced EMT process in human breast cancer cells

**Figure 3:**
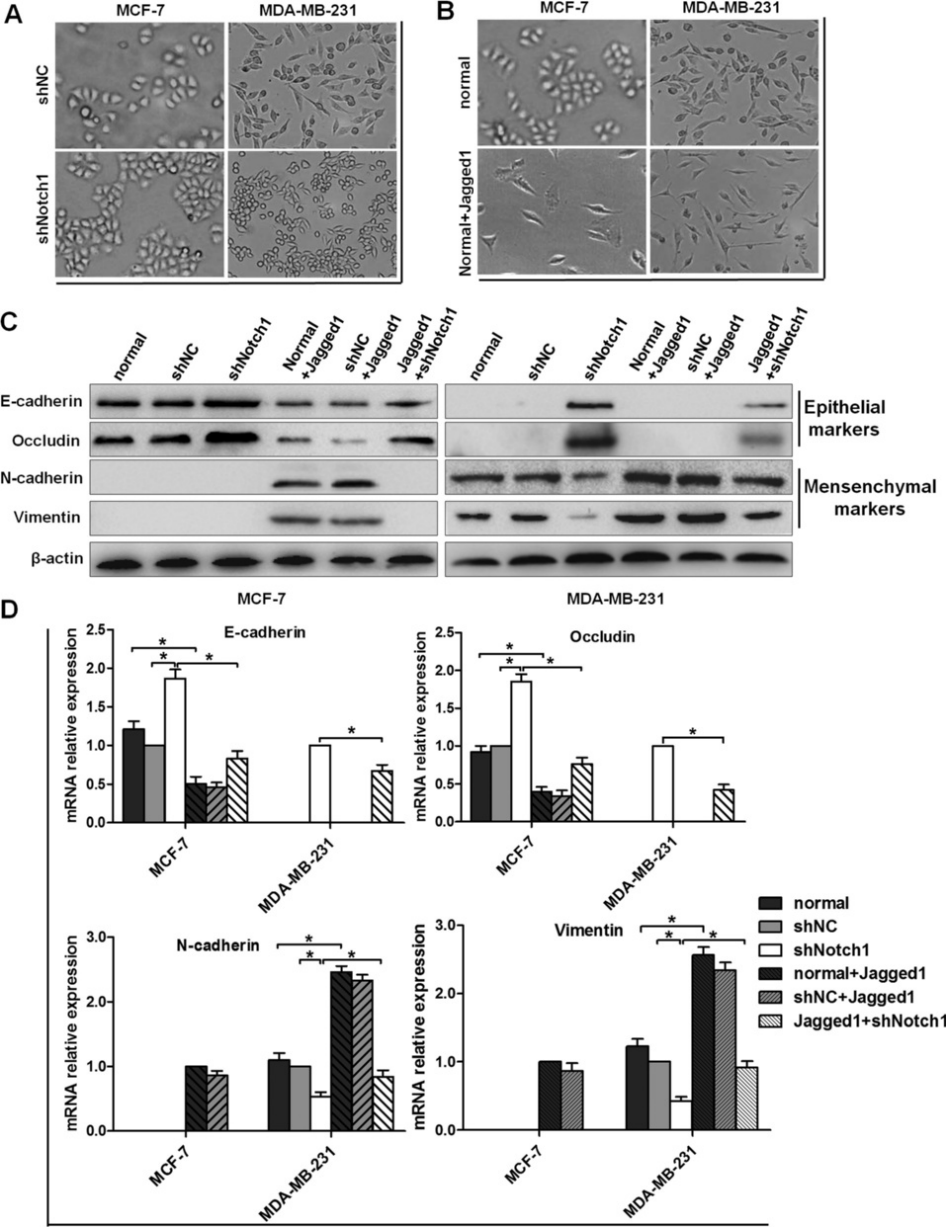
**Characteristic of EMT under conditions of Notch1 knockdown or Jagged1 induced Notch signaling activation in breast cancer cells. (A)** and **(B)** Morphological changes of MCF-7 and MDA-MB-231 cells. **(C)** Total protein was extracted for immunoblotting analysis of the EMT-related markers E-cadherin, occludin, N-cadherin, and vimentin in MCF-7 and MDA-MB-231 cells. β-Actin was used as a loading control. **(D)** Real-time PCR analysis of the EMT-related markers E-cadherin, occludin, N-cadherin, and vimentin in MCF-7 and MDA-MB-231 cells. GAPAH was used as a normalization control. The data are from three independent experiments. Column: mean; bar: SD. The symbol * represents a significant difference (P < 0.05).

EMT plays a critical role in the process of tumor migration and invasion [4]. To investigate whether Notch signaling regulates the EMT process in breast cancer cells, we established stable Notch1-shRNA-transfected cells (MCF-7-shNotch1 cells and MDA-MB-231-shNotch1 cells) to inhibit Notch1 expression. When Notch1 shRNA was stably transfected into these two cell lines, MDA-MB-231cells, which are more tumorigenic and invasive, presented an obvious morphological change, from the spindle phenotype (mesenchymal phenotype) to a rounded or cobblestone phenotype (epithelial phenotype) (Figure 3A). However, MCF-7 cells, which exhibited a typical cobblestone-shaped phenotype with less aggressive invasion ability, had no obvious morphological change (Figure 3A). In contrast, immobilized Jagged1-induced Notch activation transformed the morphology of both cells from a cobblestone epithelial phenotype to a spindle mesenchymal phenotype (Figure 3B).

**Figure 4:**
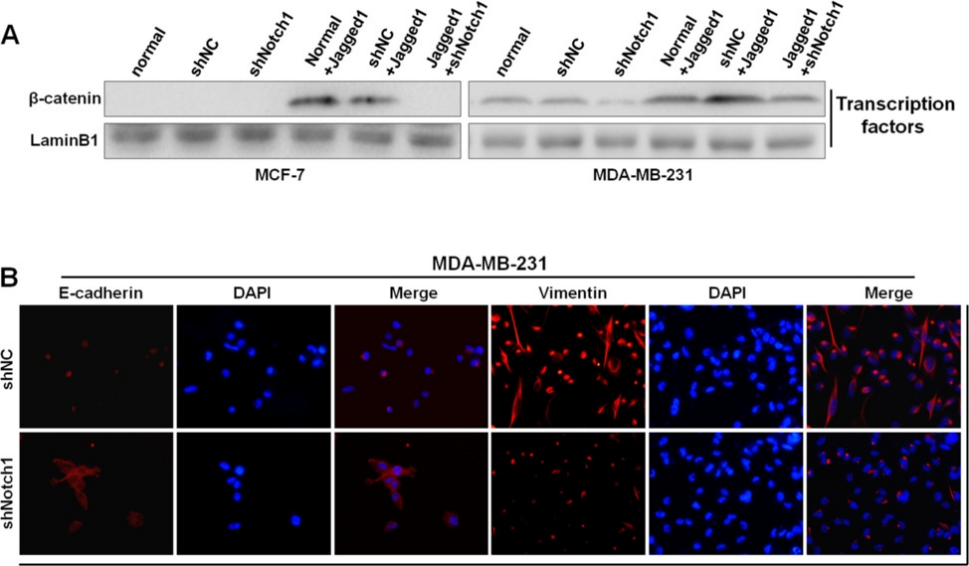
**The effects of Notch1 knockdown and Jagged1 on breast cancer EMT. (A)** Nuclear protein was extracted for western blot analysis, and a β-catenin antibody was used to observe the changes of β-catenin in the nucleus. LaminB1 was used as a loading control for nuclear lysate. **(B)** Immunofluorescence analysis of E-cadherin and vimentin in MDA-MB-231 cells. DAPI staining was used to detect nuclei and is merged with E-cadherin and vimentin in their respective panels. The cells were observed under an immunofluorescence microscope at 400× magnification. The data are from three independent experiments.

**Figure 5:**
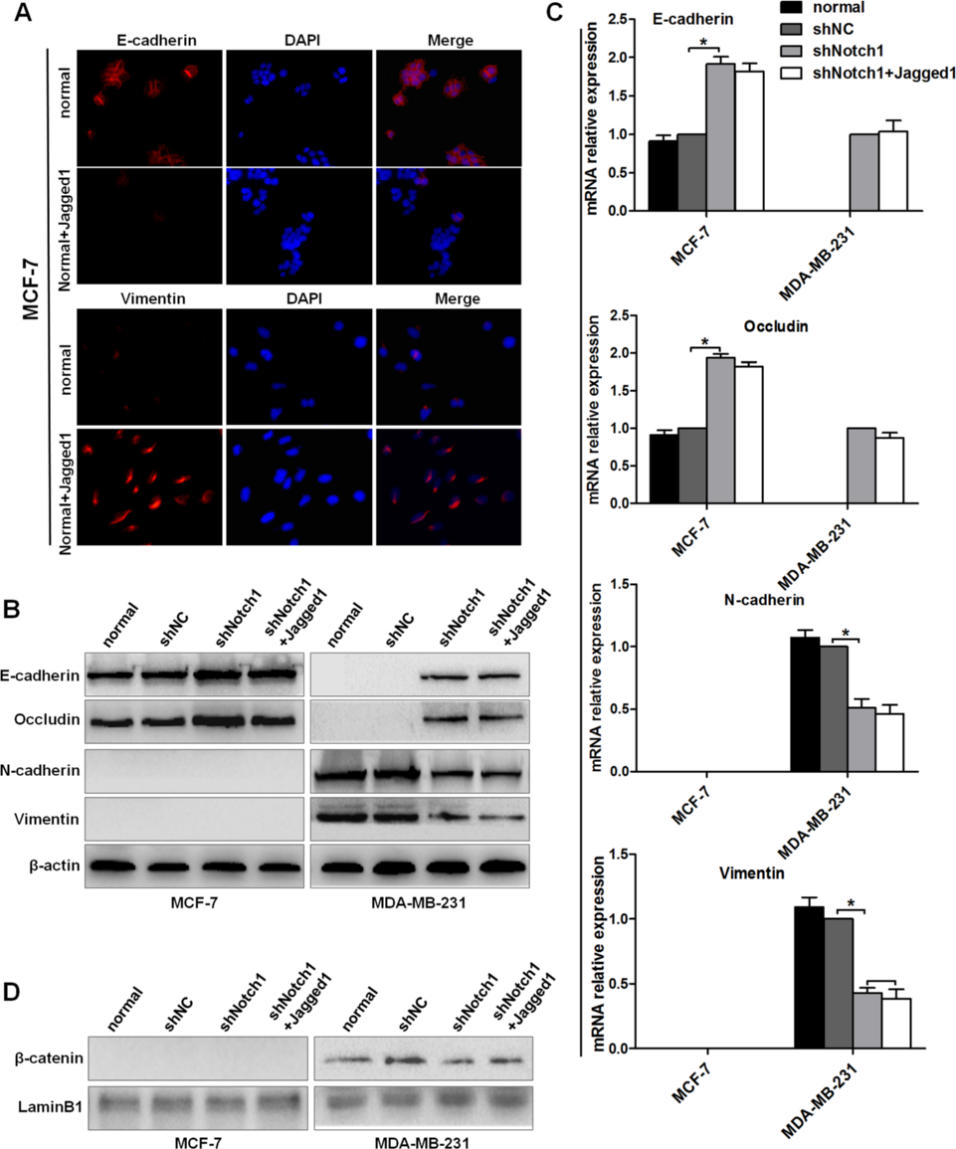
**Jagged1-mediated activation of Notch signaling induces EMT and cannot rescue the changes caused by Notch1 interference.** shNotch1 + Jagged1 group: MCF-7 or MDA-MB-231 cells were treated with Jagged1 for 48 h after stable shNotch1 transfection. **(A)** Immunofluorescence analysis of E-cadherin and vimentin in MCF-7 cells. DAPI staining was used to detect nuclei and is merged with E-cadherin and vimentin in their respective panels. The cells were observed under an immunofluorescence microscope at 400× magnification. **(B)** Western blot analysis of the EMT-related markers E-cadherin, occludin, N-cadherin, and vimentin in MCF-7 and MDA-MB-231 cells. **(C)** The mRNA expression levels of E-cadherin, occludin, N-cadherin, and vimentin were estimated by real-time PCR. **(D)** Western blot analysis of nuclear β-catenin in MCF-7 and MDA-MB-231 cells. The data are from three independent experiments. Column: mean; bar: SD. The symbol * represents a significant difference (P < 0.05).

Accompanied with the morphological changes, the protein markers associated with EMT were also changed. The expression levels of the epithelial markers E-cadherin and occludin were significantly increased in both MCF-7-shNotch1 and MDA-MB-231-shNotch1 cells (Figure 3C–D). On the contrary, the expression levels of the mesenchymal markers N-cadherin and vimentin were markedly decreased (Figure 3C–D). Meanwhile, immobilized Jagged1 ligand-induced Notch signaling activation led to a prominent decrease in the expression levels of the epithelial markers E-cadherin and occluding (Figure 3C–D) and a significant increase in the expression levels of N-cadherin and vimentin, as compared with the normal group (Figure 3C–D). In addition, nuclear protein was isolated to observe the changes of β-catenin in the cell nucleus. In the normal state, there was no expression of β-catenin in the nucleus of MCF-7 cells and only a little expression in the nucleus of MDA-MB-231 cells. Western blot assays showed that after Notch1 interference, the expression of β-catenin was not affected in the nucleus of MCF-7 cells, but it was reduced in the nucleus of MDA-MB-231 cells (Figure 4A). Conversely, immobilized Jagged1 ligand-mediated Notch signaling activation led to increased nuclear β-catenin (Figure 4A). After MCF-7 and MDA-MB-231 cells were incubated with Jagged1 for 48 h, these cells were transfected with Notch1 shRNA lentiviral vectors for another 48 h; the results showed that the changes of EMT markers induced by Jagged1 could be partially abrogated by Notch1 knockdown (Figure 3C–D; Figure 4A). Meanwhile, immunofluorescence assays in MDA-MB-231 cells confirmed that Notch1 interference significantly increased E-cadherin expression and decreased vimentin expression (Figure 4B). In addition, immunofluorescence assays in MCF-7 cells confirmed that Jagged1 significantly decreased E-cadherin expression and increased vimentin expression (Figure 5A). Taken together, our data showed that Notch1 knockdown leads to the mesenchymal–epithelial transition (MET) process, whereas Jagged1-induced Notch signaling activation promotes the EMT process in breast cancer cells.

To further investigate the correlation between Notch1 signaling and the EMT process in breast cancer, an immobilized Jagged1 ligand was used to activate stable shNotch1-transfected cells (First, the stable shNotch1-transfected cells were generated, and then stable shNotch1-transfected cells were incubated with the immobilized Jagged1 ligand for 48 h). As shown in Figure 5B–D, the effects of Notch1 interference on EMT could not be rescued by Jagged1-induced Notch signaling activation. These data indicate that Jagged1-mediated Notch signaling activation promotes the breast cancer EMT process mainly through activation of Notch1.

### Notch1 knockdown suppresses Jagged1-induced migration and invasion of breast cancer cells

**Figure 6:**
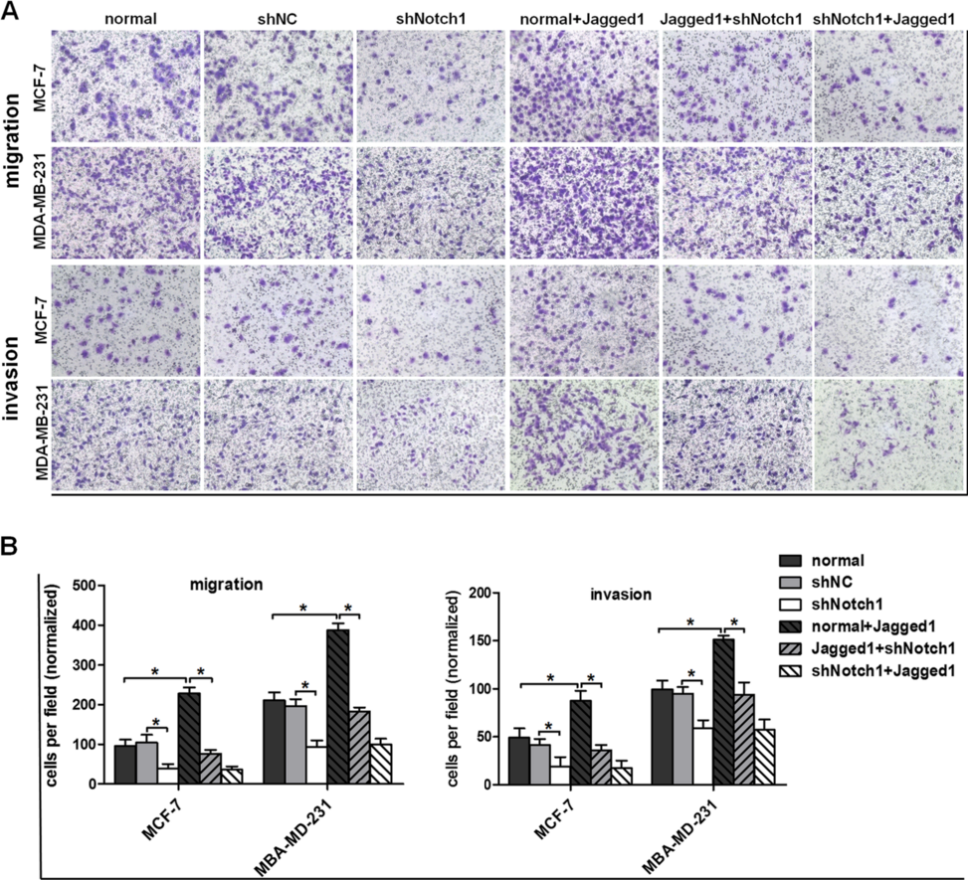
**Notch1 knockdown inhibits Jagged1-enhanced breast cancer migration and invasion. (A)** and **(B)** The cells were seeded into a migration chamber or a Matrigel-coated invasion chamber and incubated for 24 h. The number of migrated cells was quantified by counting the numbers of cells from six random fields at 100× magnification. The data are from three independent experiments. There are similar numbers of migrated and invaded breast cancer cells between the Normal + Jagged1 and the shNC + Jagged1 groups of cells (data not shown). Column: mean; bar: SD. The symbol * represents a significant difference (P < 0.05).

Next, we investigated the effect of Notch1 on the migration and invasion in MCF-7 and MDA-MB-231 cells. As shown in Figure 6A–B, Notch1 interference strongly inhibited cell migration and invasion, compared with the normal group and the shNC group in both cell lines. However, Jagged1-mediated Notch signaling activation notably enhanced the migration and invasion abilities in both cell lines.

After MCF-7 and MDA-MB-231 cells were incubated with Jagged1 for 48 h, these cells were transfected with Notch1 shRNA lentiviral vectors for another 48 h; the results showed that the enhanced migration and invasion abilities that were due to Jagged1-mediated Notch signaling activation were abolished by Notch1 silencing (Figure 6A–B). However, in the stable shNotch1-transfected cells (MCF-7-shNotch1 cells and MDA-MB-231-shNotch1 cells), after incubation with Jagged1 protein for 48 h, the inhibited migration and invasion abilities could not be restored by Jagged1-induced Notch signaling activation (Figure 6A–B), suggesting that Jagged1-induced Notch signaling activation promotes the migration and invasion mainly through activation of Notch1.

### N1ICD regulates Slug expression by inducing Slug promoter activation in breast cancer

Transcription factors, such as Snail, Slug, ZEB1, Twist, and ZEB2/SIP1, have been demonstrated to be capable of orchestrating EMT in cancer progression [10]. Therefore, we tested the expression levels of the above transcription factors with Notch1 inhibition in MDA-MB-231 human breast cancer cells. Interestingly, our results showed that with the downregulation of Notch1, the protein levels of both Slug and Snail decreased, but the Slug protein level decreased much more significantly than that of Snail. However, the protein levels of other transcription factors had no significant changes (Additional file 1: Figure S2A–B).

**Figure 7:**
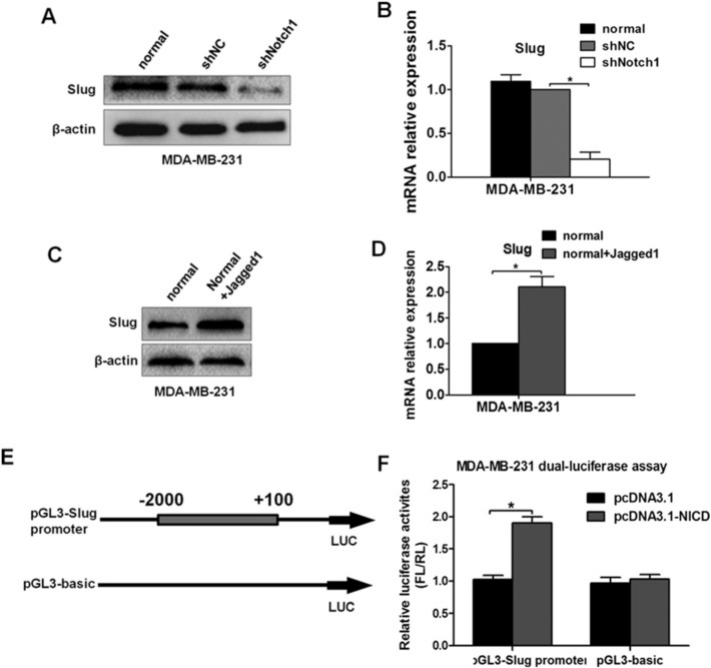
**Notch1 regulates Slug expression by enhancing its promoter activity. (A)** and **(B)** The level of Slug expression was evaluated by western blot and real-time PCR after MDA-MB-231 cells were stably transfected with shNC or shNotch1. **(C)** and **(D)** The Slug expression level following treatment with Jagged1 ligand for 48 h was estimated by western blot and real-time PCR. **(E)** The schematic diagram shows the construction of the pGL3-Slug promoter and its negative control pGL3-basic. **(F)** Luciferase reporter assays were carried out in MDA-MB-231 cells, which were cotransfected with the pGL3-Slug promoter or its negative control pGL3-basic and N1ICD overexpression plasmid pcDNA3.1(+) or its negative control plasmid pcDNA3.1. Each independent experiment was repeated three times. Column: mean; bar: SD. The symbol * represents a significant difference (P < 0.05).

Then, we examined whether Notch1 had a pivotal role in regulating Slug expression in breast cancer. As shown in Figure 7A-B, when Notch1 was silenced, both the protein level and mRNA level of Slug were significantly reduced in the Slug-positive MDA-MB-231 human breast cancer cells. Similarly, Jagged1-induced Notch signaling activation resulted in a marked increase in the expression of Slug in the MDA-MB-231 cells (Figure 7C-D). To determine whether the altered Slug expression resulted directly from the regulation of Notch1 or its downstream proteins, Hes1 and Hey1 were also knocked down with siRNAs in MDA-MB-231 cells (Additional file 1: Figure S3A–B). Our data showed that the protein expression level of Slug did not change significantly with Hes1 or Hey1 inhibition (Additional file 1: Figure S3C), suggesting that Slug is a target gene of Notch1 but not the downstream gene of Hey1 or Hes1. Based on the above data, we speculated that Notch1 might have participated in the transcriptional regulation of Slug. To validate this hypothesis, we performed a promoter assay by using the N1ICD overexpression plasmid pcDNA3.1(+) or its negative control plasmid pcDNA3.1 co-transfected with the reporter constructs of the pGL3-Slug promoter (from −2000 to +100 relative to the transcription start site) or its negative control pGL3-basic, respectively (Figure 7E). As shown in Figure 7F, N1ICD significantly increased the activity of the Slug promoter compared with the transfected control in the pGL3-Slug promoter group, but not in the pGL3-basic group. These results indicate that N1ICD regulated Slug expression by inducing Slug promoter activation at the transcription level.

### Slug serves as a mediator for Notch1-induced EMT

**Figure 8:**
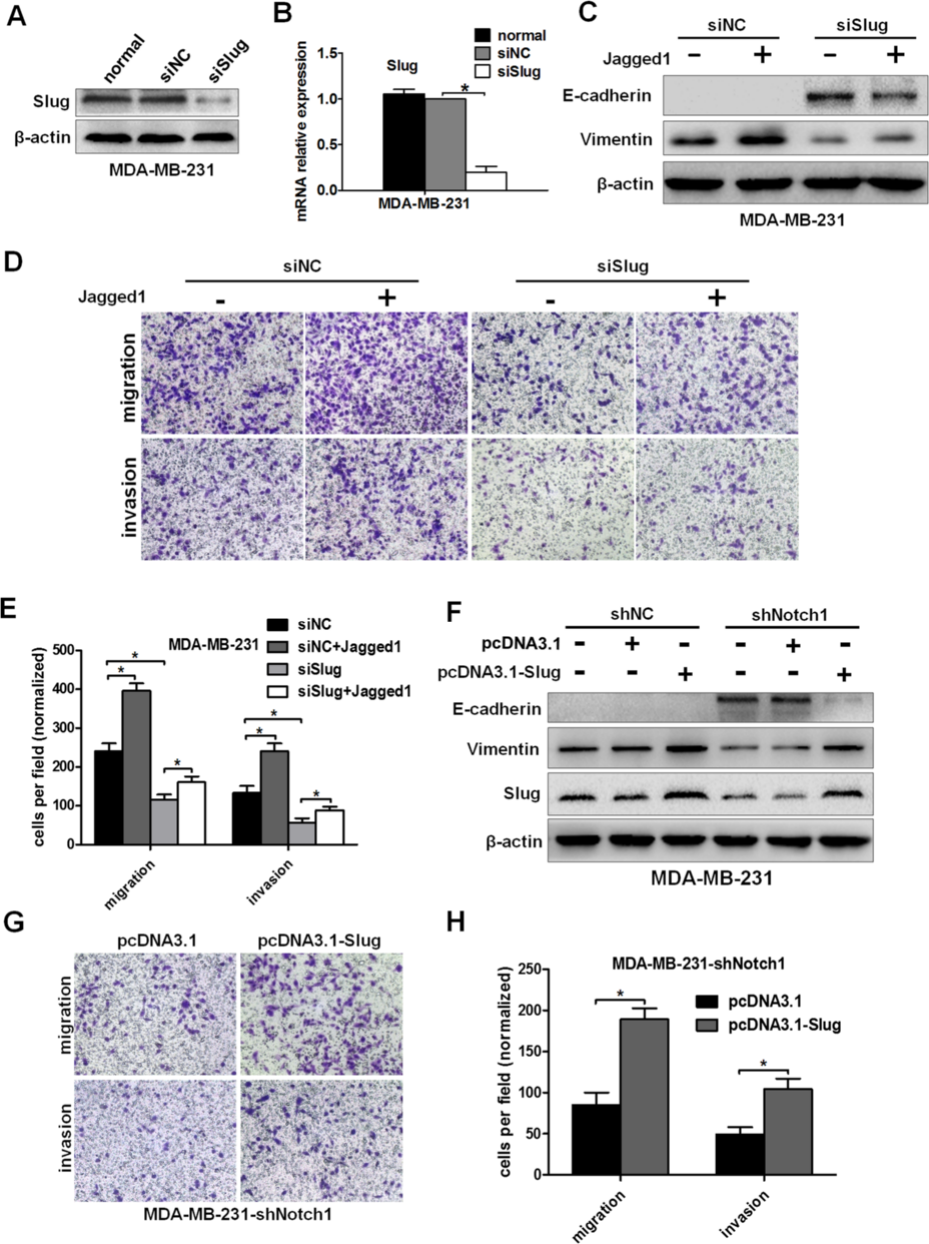
**Slug serves as a mediator for Notch1-induced EMT, migration, and invasion. (A)** and **(B)** Western blot analysis and real-time PCR assays were carried out to evaluate the expression of Slug when MDA-MB-231 cells were transfected with negative control siRNA (siNC) or Slug siRNA (siSlug) for 48 h. **(C)** MDA-MB-231 cells were transfected with siNC or siSlug for 48 h and then treated with Jagged1 for an additional 48 h. E-cadherin and vimentin protein levels were evaluated by western blot. **(D)** and **(E)** MDA-MB-231 cells were transfected with siNC or siSlug for 48 h and then treated with Jagged1 for an additional 48 h. The cells were seeded into a migration chamber or a Matrigel-coated invasion chamber and incubated for 24 h. The number of migrated cells was counted under a light microscope. **(F)** MDA-MB-231-shNC or MDA-MB-231-shNotch1 cells were transfected with negative control vector (pcDNA3.1) or Slug overexpression vector (pcDNA3.1-Slug), respectively. Forty-eight hours later, western blot analysis was performed to assess the expression levels of E-cadherin and vimentin. **(G)** and **(H)** MDA-MB-231-shNotch1 cells were transfected with pcDNA3.1 or pcDNA3.1-Slug for 48 h and then the migration and invasion abilities were evaluated. The data are from three independent experiments. Column: mean; bar: SD. The symbol * represents a significant difference (P < 0.05).

To explore whether Notch1 exerts its function through its target gene Slug, we knocked down Slug expression by Slug siRNA in MDA-MB-231 cells (Figure 8A–B) and determined the expression levels of EMT markers by western blot assays. The Slug-knocked down cells displayed increased E-cadherin and decreased vimentin expression levels. Moreover, Jagged1-mediated Notch signaling activation could partially rescue these changes (Figure 8C). We further examined the migration and invasion abilities of MDA-MB-231 cells transfected with Slug siRNA. Downregulation of Slug strongly inhibited cell migration and invasion, whereas Jagged1 could partially restore the migration and invasion (Figure 8D–E).

Next, we focused on the role of the transcription factor Slug in the Notch1-induced EMT process. If Slug expression is important to the Notch1-induced EMT, the upregulation of Slug is expected to restore the EMT changes mediated by Notch1 knockdown. To test this possibility, stable MDA-MB-231-shNC cells or MDA-MB-231-shNotch1 cells were transfected with pcDNA3.1(+)-vector or pcDNA3.1(+)-Slug for 48 h. Western blot analysis indicated that overexpression of Slug reduced the expression of the epithelial marker E-cadherin and partially restored the expression of the mesenchymal marker vimentin (Figure 8F). Moreover, Slug upregulation prominently enhanced the migration and invasion abilities of MDA-MB-231-shNotch1 cells (Figure 8G–H). These results indicated that Slug plays a key role in Notch1 signaling to regulate EMT and invasion in breast cancer cells.

### Notch1 silencing reverses EMT in breast cancer *in vivo*

**Figure 9:**
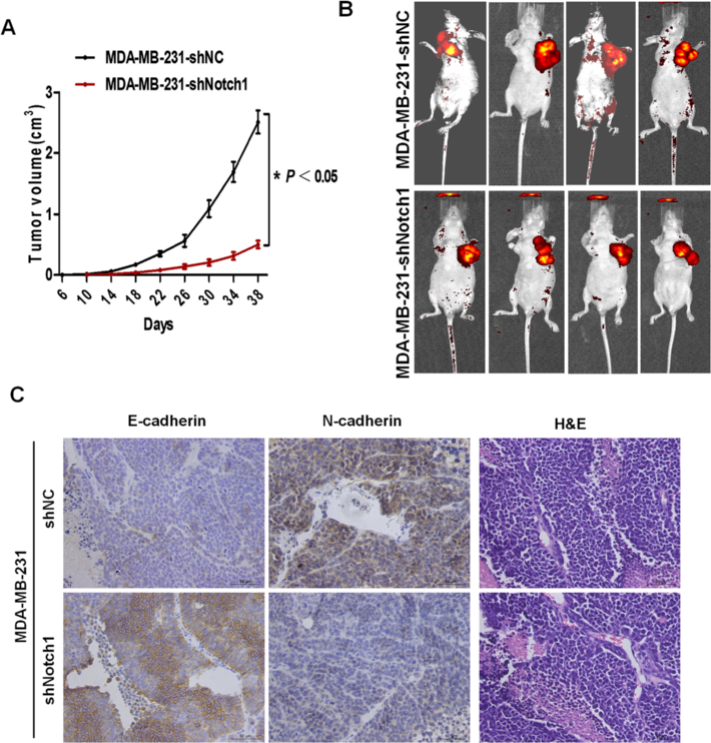
**Downregulation of Notch1 reverses EMT** ***in vivo*** **. (A)** Tumor growth curve. The tumor volume in nude mice injected with MDA-MB-231-shNotch1 cells was significantly smaller than that of the control MDA-MB-231-shNC cells. Tumor volumes represent means ± SD, n = 4 per group, the symbol * represents a significant difference (P < 0.05). **(B)** *In vivo* metastasis assays of MDA-MB-231 cells with or without Notch1 downregulation. **(C)** Representative images of immunohistochemical staining for the EMT-related markers E-cadherin and N-cadherin as well as hematoxylin and eosin staining (magnification: 400×). The data are from three independent experiments.

*In vitro* experiments have revealed that Notch1 silencing reverses the EMT process and inhibits metastasis in breast cancer cells, which prompted us to investigate whether downregulation of Notch1 can regulate EMT *in vivo*. Stable shRNA-transfected cells (MDA-MB-231-shNotch1 and MDA-MB-231-shNC) were orthotopically injected into the mammary fat pads of 6-week-old female nude mice. The tumor growth curve showed that the tumors of the MDA-MB-231-shNotch1 group grew much more slowly than those of the MDA-MB-231-shNC group (Figure 9A–B). Immunohistochemical analysis displayed a decrease in the N-cadherin level but an increase in the E-cadherin level (Figure 9C), which represents the MET phenotype.

## Discussion

In the current study, our data showed that inhibition of Notch1 reversed the EMT process both *in vitro* and *in vivo*, and inhibited migration and invasion in breast cancer cells. We found a positive association between Notch1 signaling and breast cancer invasion and progression, thus presenting a potential oncogenic role. Moreover, our study identified that EMT induced by Jagged1 is mainly through Notch1-induced Notch signaling activation and that Slug is an important target gene of Notch1 signaling in regulating the EMT process. Notch signaling, which plays a critical role in tumor pathology and progression, is frequently observed in breast cancer and other solid tumors [28]. Recently, several studies have revealed that overexpression of Notch1 and/or Jagged1 indicates a poor prognosis for breast cancer patients. Furthermore, the Notch signaling pathway is related to a number of protumorigenic activities in breast cancer cell lines and can cause mammary hyperplasia and carcinogenesis in mice [29-32]. A study on human breast cancer has shown that the protein level of Numb, which is a negative regulator of Notch signaling, is reduced in 50% of human breast tumors [33]. Notch1 has also been found to be a downstream effector of the oncogenic gene Ras in human mammary tumorigenesis [34]. Although there is abundant evidence demonstrating that the Notch signaling pathway is closely associated with human breast cancer development, our study has provided novel insights into the role of Notch1 signaling in regulating EMT and invasion mainly in a Slug-dependent manner.

Invasion and metastasis are two of the most important features of malignant tumors, the main cause of cancer-related death, and the most difficult problem in clinical treatment. EMT is a key step in tumor invasion and metastasis. Our study results emphasize the key role of the Notch1 signaling pathway in regulating EMT via the transcription factor Slug during cancer cell invasion and metastasis, using both *in vitro* and *in vivo* models. Our data showed that the expression of the Notch signaling downstream genes Hes1 and Hey1 decreased in breast cancer cells with Notch1 inhibition. In contrast, the expression levels of Hes1 and Hey1 were significantly increased during Notch signaling pathway activation induced by Jagged1. These data indicate that both Hes1 and Hey1 are sensitive Notch target genes for the Notch signaling pathway. While previous studies have shown that both Hes1 and Hey1 are sensitive Notch target genes, *Hey* is more sensitive than *Hes* gene for Notch pathway inhibition in breast cancer [35]. Recently, a few other Notch target genes have been found, including NF-κB, cyclinD1, c-myc, p21, p27, Akt, mTOR, VEGF, etc. [20,21,36]. In this study, our data also showed that NF-κB p65 expression was dramatically downregulated by Notch1 knockdown in breast cancer cells. Similarly, a recent report has revealed that Notch signaling pathway inhibition results in decreased NF-κB in pancreatic cancer cells, suggesting a molecular link or cross-talk between Notch and NF-κB; furthermore, activation of these signaling pathways is associated with acquisition of the EMT phenotype [37].

Previous studies have demonstrated that Jagged1-induced Notch signaling activation induces EMT through Slug-mediated repression of E-cadherin [35]. A recent study has shown that Notch1 mediates esophageal carcinoma cell invasion and metastasis by inducing EMT through upregulation of Snail [38]. Likewise, our study showed that the EMT process was closely related to Notch1 signaling in breast cancer cells. Notch1 silencing could reverse the EMT process and lead to MET. The MET program is characterized by increased E-cadherin and occludin expression, and decreased N-cadherin, vimentin, and nuclear β-catenin, which leads to the acquisition of an epithelial phenotype. Meanwhile, breast cancer cell migration and invasion abilities were inhibited by Notch1 interference. Furthermore, our data showed that the EMT process was largely driven by Jagged1-induced Notch signaling activation. This EMT program displayed a significant reduction of E-cadherin and occludin expression, and a marked increase of N-cadherin, vimentin, and nuclear β-catenin expression, resulting in cancer cells evolving into a highly invasive and mesenchymal phenotype. A recent study has reported that EMT results in a loss of E-cadherin, which impairs cell–cell adhesion and allows nuclear localization of β-catenin [39]. Apparently, our findings are in concordance with this study. Notably, culturing breast cancer cells with soluble Jagged1 protein induced EMT and increased their migration and invasion, which could be partially abolished by Notch1 knockdown. These data indicate that the Jagged1–Notch1 axis plays an important role in promoting EMT and invasion in breast cancer. In addition, Chen *et al.* have shown that inhibition of Notch signaling abrogates the downregulation of E-cadherin and increases migration and invasion under hypoxic conditions in breast cancer [12]. Interestingly, our results showed that Jagged1 could not rescue the changes caused by downregulation of Notch1. Jagged1 ligand-triggered Notch activation is mainly mediated by the interaction between Notch ligands and receptors. Our data also showed that Jagged1 could cause a significant upregulation of N1ICD and less upregulation of N4ICD. Our study provides noteworthy evidence that the Jagged1 ligand can interact with Notch1 to activate Notch signaling and promote EMT.

Slug and Snail are associated with EMT during both embryonic development and cancer metastasis [40,41]. Our data showed that Notch1 knockdown mainly decreased Slug expression, compared with the other transcription factors. Downregulation of Slug resulted in a significant increase of E-cadherin expression and a prominent decrease of the vimentin level, and weakened the migration and invasion capacities in breast cancer cells. Importantly, the Notch1-reversed EMT was restored by the overexpression of Slug. Furthermore, Notch1 was found to be more effective at downregulating the Slug expression levels than Hes1 and Hey1. This result led us to investigate whether Slug expression was directly regulated by Notch1 at the transcriptional level. The luciferase reporter assay further demonstrated that Notch1/NICD positively regulated Slug expression by enhancing Slug promoter activity. In light of our studies, Notch1 might downregulate Slug to maintain the epithelial phenotype and inhibit the migration and invasion behavior of breast cancer cells. The Notch1–Slug axis might play an important role in breast cancer progression. However, the detailed mechanism by which the Notch1–Slug axis regulates EMT remains unclear, and much work needs to be done in the future.

In our experiments, we also observed that Notch signaling activation induced by Jagged1 partially inhibited the upregulation of E-cadherin and the downregulation of vimentin caused by Slug interference. These data suggest that Notch signaling could bypass Slug to trigger some other factors to regulate EMT. Leong *et al.* have reported that Notch signaling activation targets Slug, not Snail or Twist, to suppress E-cadherin and initiate the EMT process [35]. However, Chen *et al*. have shown that Notch signaling activation under hypoxic conditions could increase the expression of Slug and Snail to initiate EMT [12].

Taken together, our observations imply that Jagged1-triggered Notch signaling activation is mainly dependent on Notch1. Slug serves as a mediator, which contributes to the enhanced migration and invasion in breast cancer cells. EMT is initiated mainly though the Notch1–Slug signaling axis. Thus, Slug plays a key role in Notch1 signaling that modulates EMT and metastasis in breast cancer.

## Conclusion

Tumor metastasis is a main cause of mortality in cancer patients. EMT favors tumor metastasis and recurrence. Notch1 knockdown increased E-cadherin expression and reversed EMT *in vitro* and *in vivo*, and inhibited the motility and invasion capacities of breast cancer cells. In addition, the Notch1–Slug axis was shown to be vital for the Jagged1-induced promotion of EMT and invasion in breast cancer. Here, we provide evidence for the Notch1–Slug signaling axis in breast cancer cells to promote EMT and further enhance the capacity of migration and invasion, which may improve our understanding of the regulatory networks governing EMT and cancer progression. Our results highlight the potential use of Notch1 signaling or Slug inhibitors to prevent breast cancer progression.

## Materials and methods

### Cell culture

The human breast cancer cell lines (MCF-7, MDA-MB-231, SK-BR-3, T47D, and ZR-75-1) used in this study were purchased from the Cell Bank of Type Culture Collection of the Chinese Academy of Sciences (Shanghai, China). Human mammary epithelial cells (HMECs) and non-tumorigenic MCF-10A cells were obtained from the American Type Culture Collection. The human breast cancer cells were cultured in DMEM, RPMI-1640, or McCoy’s 5A medium, respectively, according to the recommended culture method. All media were supplemented with 10% fetal bovine serum (FBS; Gibco, Grand Island, NY, USA) and 1% penicillin-streptomycin. All cells were grown at 37°C in an atmosphere of 5% CO_2_. The culture methods used for HMECs and MCF-10A cells were as described previously [42].

### Notch signaling activation by using immobilized recombinant Notch ligands

The Notch signaling activation assay was performed as described previously [43]. Cell culture plates were coated with 50 μg/ml Protein G (Zymed, USA) in phosphate-buffered saline (PBS) at room temperature overnight. The plates were washed with PBS three times and blocked with 10 mg/ml bovine serum albumin (BSA) in PBS for 2 h at room temperature. The blocked plates were then washed with PBS and incubated with recombinant Jagged1-FC chimera (R&D Systems, USA) at a concentration of 3 μg/ml in 0.1% BSA/PBS for 3 h at room temperature. After washing three times with PBS, the cells were immediately seeded in the coated plates using the culture medium as described above. The schematic diagram of this Notch signaling activation method is shown in Additional file 1: Figure S4.

### Notch1 shRNA lentivirus transfection

Notch1 shRNA (shNotch1) and negative control (shNC) in eukaryotic GV248 lentiviral vectors were purchased from GeneChem Co., Ltd. (Shanghai, China). The target sequence for Notch1 shRNA was *GTCCAGGAAACAACTGCAA.* The cells were seeded at 1 × 10^3^ cells/well into 96-well plates at 24 h prior to transfection. When the cells grew to 30–70% confluence, transfection was carried out by using lentiviral particles (MCF-7 multiplicity of infection (MOI) = 20; MDA-MB-231 MOI = 10), polybrene (5 μg/ml), and enhanced infection solution (GeneChem Co., Ltd., Shanghai, China), according to the manufacturer’s protocol. At 12 h post-transfection, virus-containing medium was replaced with complete medium. At 96 h post-transfection, all cells were selected by puromycin (Merck, USA) at a final concentration of 5 μg/ml (MCF-7) or 4 μg/ml (MDA-MB-231) for 10 days. Then, the cells were maintained in 2.5 μg/ml (MCF-7) or 2 μg/ml (MDA-MB-231) puromycin. To generate stable transfected cells, 100 transfected cells were seeded into a 10-cm Petri dish, and the medium was changed three times per week. After 3 weeks, puromycin-resistant colonies were isolated and seeded into 96-well plates for further study. The 954-bp coding sequence of Slug was amplified by PCR from the cDNAs of MDA-MB-231 cells and subcloned into pcDNA3.1 (+) by EcoR V and EcoR I (Shanghai Genechem Co., Ltd, China).

### RNA interference

The siRNAs for Slug (*5′-GCAUUUGCAGACAGGUCAAdTdT-3′*, *5′-UUGACCUGUCUGCAAAUGCdTdT-3′*), Hey1 (*5′-CAGUUUGUCUGAGCUGAGATT-3′*, *5′-UCUCAGCUCAGACAAACUGTT-3′*), Hes1 (*5′-AGGCUGGAGAGGCGGCUAATT-3′*, *5′-UUAGCCGCCUCUCCAGCCUTT-3′*), and negative control (NC: *5′-UUCUCCGAACGUGUCACGUTT-3′*, *5′-ACGUGACACGUUCGGAGAATT-3′*) were synthesized by GenePharm (Shanghai, China). Cells (5 × 10^4^ per well) were seeded in six-well plates 24 h prior to transfection and transfected with 80 pM siRNA using TurboFectTM siRNA Transfection Reagent (Fermentas, Beijing, China), according to the manufacturer’s protocol.

### Western blot analysis and antibodies

The cells were harvested, and total protein was extracted from the stable cell lines. Nuclear protein was isolated according to the manufacturer’s instructions (Pioneer Biotechnology, Inc.). Equal amounts of protein (150 μg) were separated by sodium dodecyl sulfate-polyacrylamide gel electrophoresis (10% SDS-PAGE) and then transferred onto a polyvinylidene difluoride (PVDF) membrane (Roche). The immunoblots were incubated in 5% (w/v) skim milk powder dissolved in TBST (10 mM Tris–HCl, pH 8.0, 150 mM NaCl, and 0.05% Tween-20) for 2 h at room temperature. Next, the blots were probed first with specific antibodies and then with the appropriate secondary antibodies.β-Actin or LaminB1 was used as a control. Signals were quantified using Image-Pro Plus 6.0 software (Media Cybernetics).

Antibodies were purchased from the following sources: anti-Notch1 antibody (Cell Signaling Technology, Boston, MA, USA), anti-NF-κB65 antibody (Cell Signaling Technology), anti-Hey1 antibody (Abcam, Cambridge, MA, USA), anti-Hes1 antibody (Cell Signaling Technology), anti-E-cadherin antibody (Cell Signaling Technology), anti-N-cadherin antibody (Abcam), anti-vimentin antibody (Cell Signaling Technology), anti-occludin antibody (Proteintech Group Inc., USA), anti-Slug antibody (Abcam), anti-β-catenin antibody (Cell Signaling Technology), anti-Snail (Proteintech Group Inc.), anti-Twist antibody (Proteintech Group Inc.), anti-ZEB1 (Cell Signaling Technology), anti-ZEB2 (Abcam), anti-β-actin antibody (Santa Cruz Biotechnology, Santa Cruz, CA, USA), anti-LaminB1 antibody (Santa Cruz Biotechnology), anti-N2ICD (Santa Cruz Biotechnology), anti-N3ICD (Santa Cruz Biotechnology), and anti-N4ICD (Santa Cruz Biotechnology).

### RNA extraction and quantitative real-time PCR

Total RNA was isolated from breast cancer cells using TRIzol reagent (Invitrogen, CA, USA). cDNA was synthesized by using a PrimeScript RT reagent kit (Fermentas, Beijing, China). The real-time PCR was carried out using a SYBR Green PCR Kit (TaKaRa, Dalian, China), according to the manufacturer’s instructions. GAPDH was used as an internal control, and the relative expression levels were assessed using the ΔΔCt method. The sequences of the primers for real-time PCR are listed in Additional file 1: Table S1.

### *In vitro* cell migration and invasion assays

In the cell migration assay, equal numbers of cells (5 × 10^4^ cells/well) were suspended in the top compartment of a 24-well chamber (Millipore Co., Billerica, MA, USA) in 300 μl of DMEM (MCF-7 cells) or RPMI 1640 (MDA-MB-231 cells) containing 0.1% BSA. Then, 600 μl of DMEM containing 15% FBS or RPMI 1640 containing 10% FBS was added into the bottom compartment. The cells were incubated for 24 h. Then, the nonmigrated cells were removed from the membrane of the top compartment with a cotton swab, and the cells that had migrated through the membrane were fixed and stained in 4% paraformaldehyde and 0.01% crystal violet solution. The numbers of cells that migrated were determined from six random high-power fields (HPFs) visualized at 100× magnification, and means were obtained for statistical analysis.

The invasion assays were performed in a similar manner as the migration assays, except that the cells were placed in the upper compartment with a Matrigel (BD Biosciences, San Jose, CA, USA)-coated membrane, as previously described [44]. The numbers of invading cells were quantified from six random HPFs visualized at 100× magnification.

### Immunofluorescence

A total of 2 × 10^4^ cells per well were grown on glass coverslips in a 24-well plate overnight. The next day, when the cells were 50–70% confluent, they were washed twice with PBS, then fixed in 4% paraformaldehyde solution, and permeabilized in 0.03% Triton X-100 (Sigma) in PBS for 20 min. The cells were then washed three times (5 min each time) with PBS and blocked with 5% BSA in PBS for 1 h at room temperature. The cells on the coverslips were incubated in a humidified box with the respective primary antibodies at a 1:100 dilution overnight at 4°C. Next, the cells were washed three times (5 min each time) in PBS and incubated for 1 h with CY3-conjugated secondary antibodies at a 1:50 dilution (CWBIO, Beijing, China) at room temperature in the dark. Finally, the cells were washed three times in PBS and incubated with 1 μg/ml 4, 6-diamidino-2-phenylindole (DAPI, Roche) for 5 min at room temperature in the dark. Then, the slides were washed extensively with PBS and observed with an immunofluorescence microscope (Nikon, Japan) with identical exposure times at 400× magnification.

### Luciferase reporter assays

The cDNA encoding N1ICD was subcloned into the expression vector pcDNA3.1(+) between the BamHI and XhoI sites, and subcloning was confirmed with sequencing by Shanghai Genechem Co., Ltd. The pGL3 reporter construct plasmid (−2000/+100) consisted of a 2100-bp genomic DNA fragment of the Slug promoter (Promega, Madison, WI, USA). The pGL3-Slug promoter plasmid or its negative control pGL3-basic plasmid carrying the firefly luciferase reporter were co-transfected with an internal control, pRL-TK Renilla vector (Promega), by using Lipofectamine 2000 (Invitrogen). In addition, cells were respectively transfected with 600 ng of N1ICD overexpression plasmid pcDNA3.1(+) or its negative control pcDNA3.1. Cell lysates were harvested 48 h after transfection. The firefly and renilla activities were measured by the Dual-Luciferase Reporter Assay System (Promega). Firefly luciferase activity was normalized to the Renilla luciferase activity. Each transfection was repeated three times.

### Animal studies

Animal experiments were performed in accordance with the Animal Care and Use Committee guidelines of Xi’an Jiao Tong University, Shaanxi, China. The mice were divided into two groups, with four per group. The cells were resuspended in a 1:1 (v/v) mixture of culture media and Matrigel (BD Biosciences, San Jose, CA, USA), and 2 × 10^6^ MDA-MB-231 cells were orthotopically injected into the mammary fat pads of 6-week-old female nude mice (Silaike Laboratory Animal Co., Ltd., Shanghai, China). Tumor growth was observed twice per week with calipers at the site of injection. After 38 days, the animals were observed by an IVIS imaging system (IVIS spectrum, Xenogen, CA, USA), then they were sacrificed, and the mammary tumors were isolated for immunohistochemical staining.

### Immunohistochemistry

Sections of paraffin-embedded, formalin-fixed tumor tissues were deparaffinized in xylene and rehydrated in a series of graded ethanol solutions, and exposed to microwave radiation in a citrate buffer (pH = 6.0) for 15 min. Endogenous peroxidase activity was blocked with 3% H_2_O_2_ in methanol for 5 min at room temperature. The sections were washed three times with PBS and incubated with goat serum for 20 min at room temperature. Next, the sections were incubated with the primary antibodies overnight at 4°C. The sections were warmed to room temperature, then washed three times with PBS, incubated with biotinylated secondary antibodies for 30 min at room temperature, and washed again, after which immune complexes were detected with the use of a streptavidin-peroxidase complex (DAKO) and 3,3′-diaminobenzidine (DAB, DAKO). The sections were counterstained with hematoxylin, dehydrated in a graded series of alcohol solutions, and mounted in Malinol (Muto Pure Chemicals).

### Statistical analysis

All data are presented as the mean ± standard deviation (SD). Data were analyzed by one-way analysis of variance (ANOVA) or two-way ANOVA as appropriate. Statistical analysis was performed with SPSS 13.0 for Windows software (SPSS Inc., Chicago, IL, USA). All statistical tests were two-sided, and P values less than 0.05 were considered statistically significant. In all figures, (*) denotes P < 0.05. All experiments were repeated independently at least three times.

## Additional file

Additional file 1: **Supplementary materials.**
