# Supplementary material for: Notch1 signaling regulates the epithelial–mesenchymal transition and invasion of breast cancer in a Slug-dependent manner
Source: Mol Cancer. 2015 Feb 3;14(1):28. doi: 10.1186/s12943-015-0295-3 (PMC4322803; doi:10.1186/s12943-015-0295-3)
Supplement: Supplementary file 1 — Supplementary materials. [file 12943_2015_295_MOESM1_ESM.doc]

Supplementary materials

Figure S1


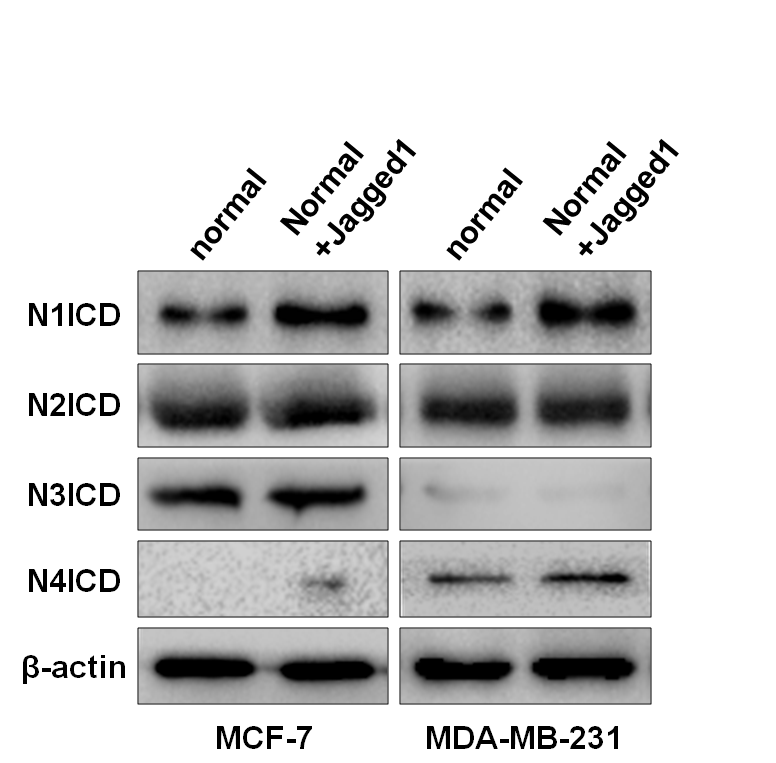


**Fig. S1 The effect of Jagged1-induced Notch signaling activation on Notch NICD.** Both MCF-7 and MDA-MB-231 cells were incubated with Jagged1 for 48 h, then the protein expression levels of Notch NICD (N1ICD, N2ICD, N3ICD, and N4ICD) were evaluated by western blot. β-Actin was used as a loading control.

Figure S2


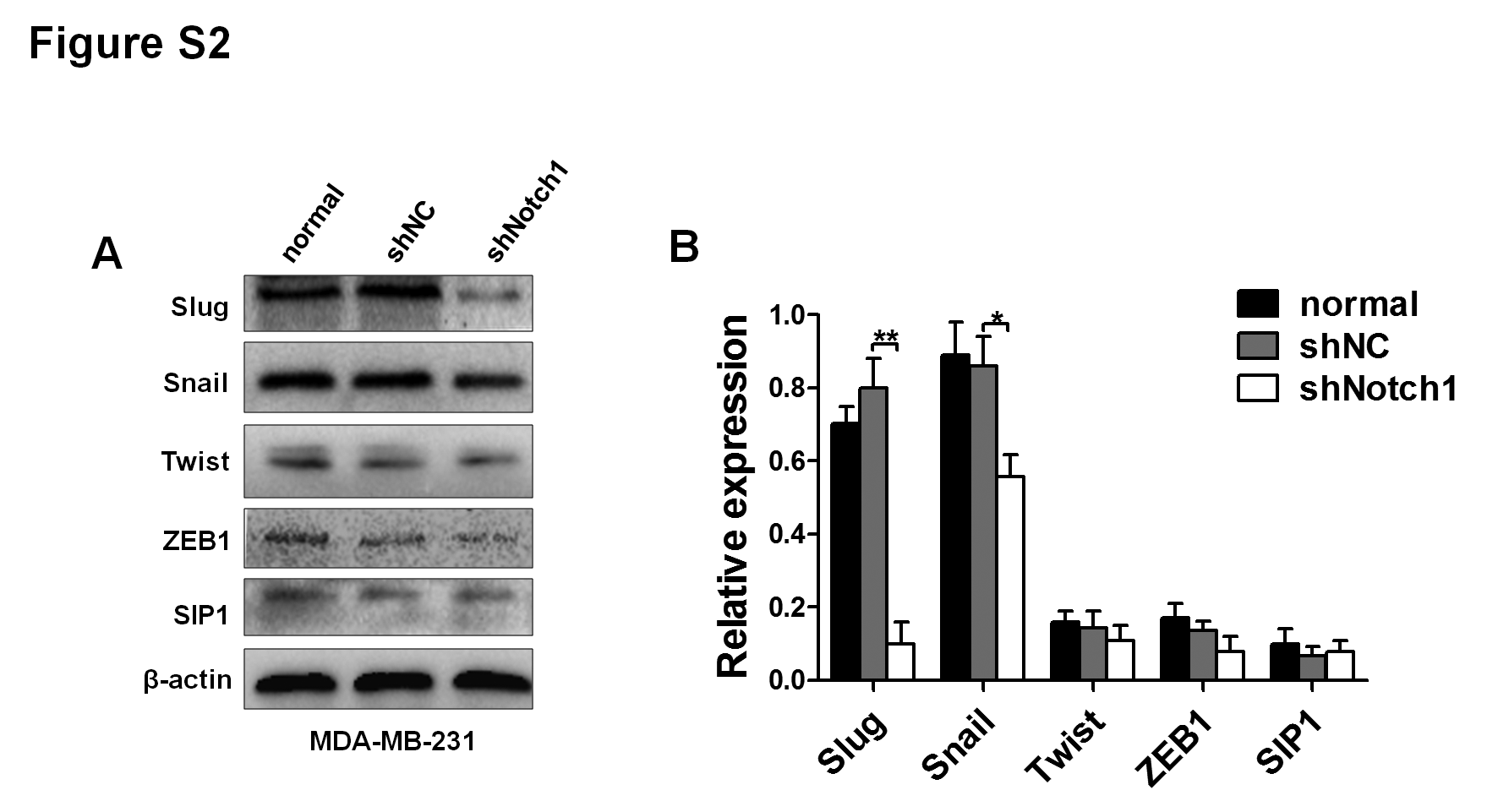


**Fig. S2 The effect of Notch1 knockdown on the transcriptional factors Slug, Snail, Twist, ZEB1, and ZEB2/SIP1 in breast cancer.** (A) The expression levels of the transcriptional factors Slug, Snail, Twist, ZEB1, and ZEB2/SIP1 were evaluated by western blot in MDA-MB-231 cells transiently transfected with shNotch1 or shNC for 48 h. β-Actin was used as a loading control. (B) The relative expression of Slug, Snail, Twist, ZEB1, and ZEB2/SIP1 at the protein levels. The data are from three independent experiments. Column: mean; bar: SD. The symbol * represents a significant difference (P < 0.05), while ** represents a highly significant difference (p < 0.001).

Figure S3


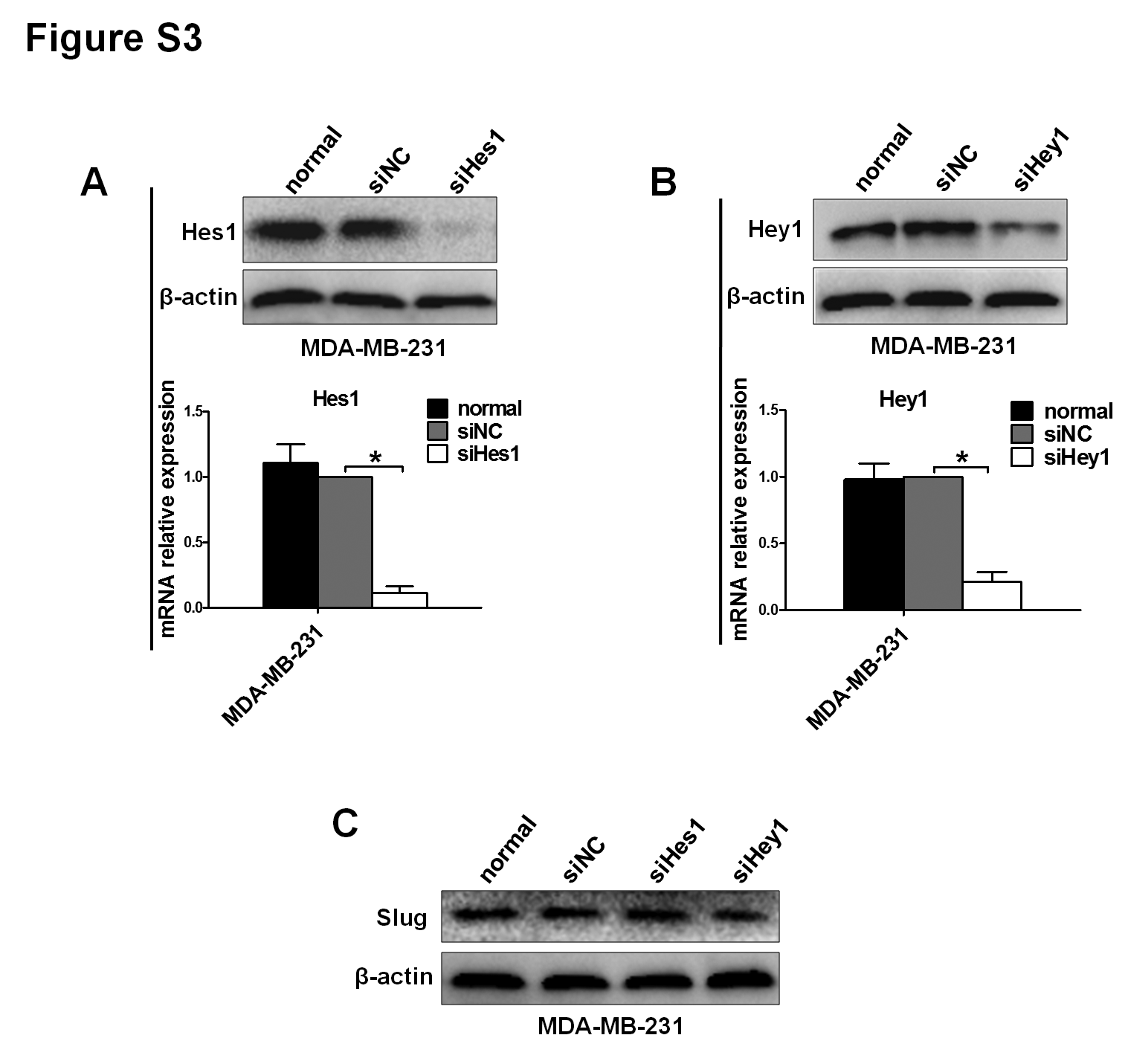


**Fig. S3 Downregulation of Hes1 and Hey1 in breast cancer MDA-MB-231 cells.** (A) and (B) Decreased expression of endogenous Hes1 or Hey1 due to knockdown of Hes1 or Hey1 in MDA-MB-231 cells. Western blot analysis and real-time PCR assay were carried out to evaluate the expression of Hes1 or Hey1 when MDA-MB-231 cells were transfected with negative control siRNA (siNC), Hes1 siRNA (siHes1), or Hey1 siRNA (siHey1) for 48 h. β-Actin was used as a loading control. (C) The Slug expression level was evaluated by western blot analysis when MDA-MB-231 cells were transfected with negative control siRNA (siNC), Hes1 siRNA (siHes1), or Hey1 siRNA (siHey1) for 48 h. The data are from three independent experiments. Column: mean; bar: SD. The symbol * represents a significant difference (P < 0.05).

Figure S4


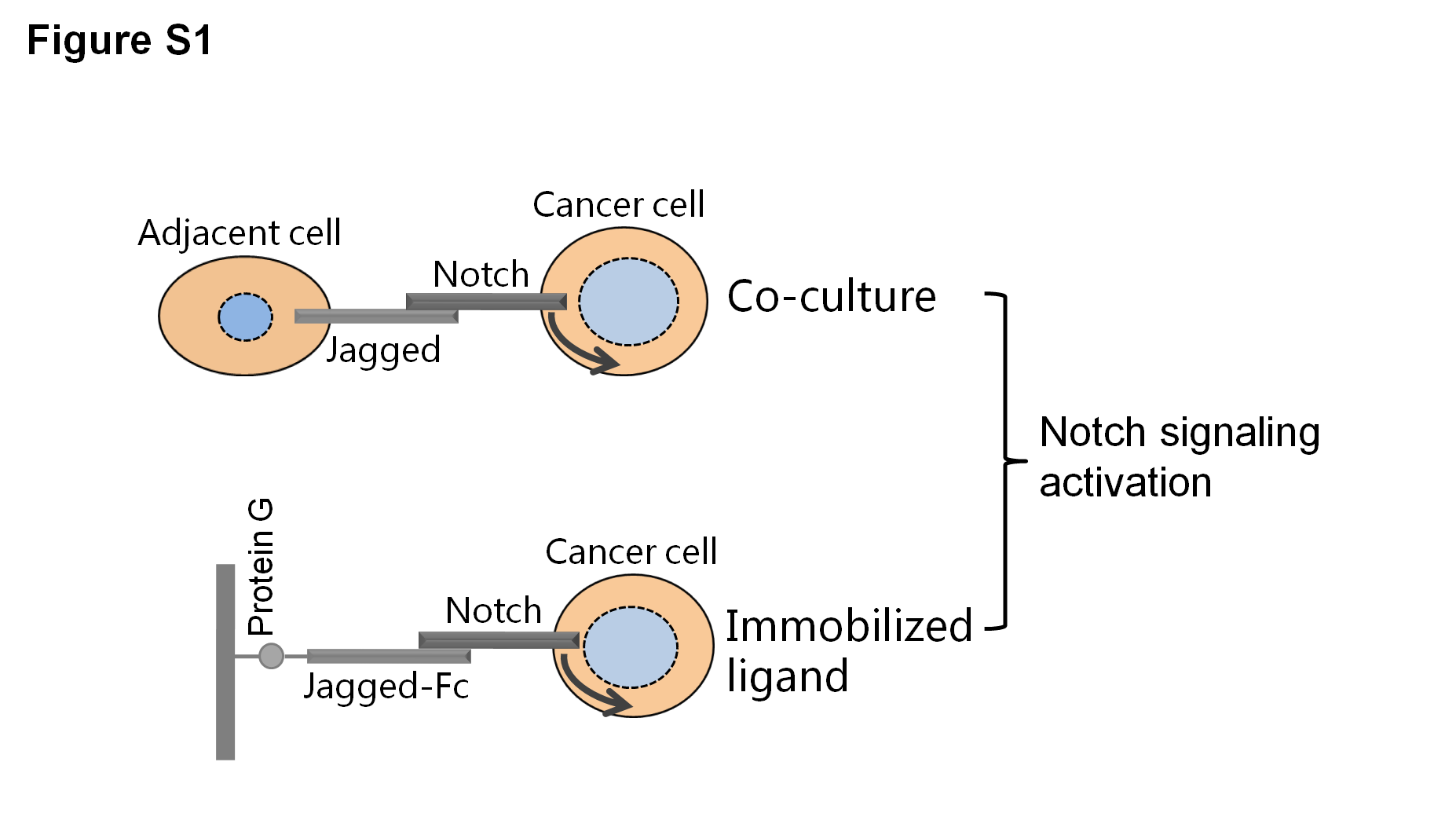


**Fig. S4** **Schematic representation of the process of Notch signaling activation in cancer cells.** There are two ways for Notch signaling activation. First, cancer cells were cocultured with cells expressing the Notch ligand Jagged1 (upper panel). Second, cancer cells were cultured on recombinant Jagged1-Fc proteins that were immobilized on cell culture plates by protein G (lower panel).

**Table S1** Primers for real-time PCR

| **Gene** | **Forward primer** | **Reverse primer** |
| --- | --- | --- |
| Notch1 | 5′- GGACCAGATTGGGGAGTT -3′ | 5′- CACACTCGTCCACATCGT -3′ |
| Hey1 | 5’-CGAGGTGGAGAAGGAGAGTG -3’ | 5’- CTGGGTACCAGCCTTCTCAG -3’ |
| Hes1 | 5′-AAGGCGGACATTCTGGAAA -3′ | 5′- AAGCGGGTCACCTCGTTCA-3′ |
| E-cadherin | 5′- ATTCTGATTCTGCTGCTCTTG-3′ | 5′-AGTCCTGGTCCTCTTCTCC -3′ |
| Vimentin | 5′-AATGACCGCTTCGCCAAC -3′ | 5′-CCGCATCTCCTCCTCGTAG -3′ |
| N-cadherin | 5′-ATGGTGTATGCCGTGAGAAG -3′ | 5′-TGTGCTTACTGAATTGTCTTGG-3′ |
| Occludin | 5′- AAGCAAGTGAAGGGATCTGC-3′ | 5′-GGGGTTATGGTCCAAAGTCA -3′ |
| Slug | 5′- AGATGCATATTCGGACCCAC -3′ | 5′- CCTCATGTTTGTGCAGGAGA -3′ |
| GAPDH | 5′-ACCACAGTCCATGCCATCAC -3′ | 5′- TCCACCACCCTGTTGCTGTA -3′ |
| NF-κB p65 | 5′-GGGAAGGAACGCTGTCAGAG-3′ | 5′-TAGCCTCAGGGTACTCCATCA-3′ |

**Table S2** Primers for expressing vectors and luciferase reporter plasmids

| Name | Sequence (5ʹ–3ʹ) |  |  |
| --- | --- | --- | --- |
| Notch1 NICD-F | TTGGATCCAAGCGCCGGCGGCAGCATG |  |  |
| Notch1 NICD-R | CGCCTCGAGCTTGAAGGCCTCCGGAATGCG |  |
| Slug-F | ATAGAATTCCCAGACCCGCTGGCAAGATG |  |  |
| Slug-R  Slug promoter-F  Slug promoter-R | AATGGATCCCGAGTAAACATTGATTGCGTCA  ATTAATCACCCTCGGATACCTGCTGAT  GGAATTCCTCCTTTACGAACTGAGCCCGT |  |  |
